# Cochlear Amyloid-β42 Accumulation Drives Progressive Auditory Neuropathy in 5XFAD Mice: A Potential Biomarker for Early Alzheimer’s Disease

**DOI:** 10.21203/rs.3.rs-6431143/v1

**Published:** 2025-04-21

**Authors:** Dheyaa Al-Sallami, Raheem F. H. AL Aameri, Shelley Tischkau, Leonard P. Rybak, Vickram Ramkumar

**Affiliations:** Southern Illinois University School of Medicine; Southern Illinois University School of Medicine; Southern Illinois University School of Medicine; Southern Illinois University School of Medicine; Southern Illinois University School of Medicine

**Keywords:** Alzheimer’s disease (AD), Hearing loss and dementia, Amyloid-beta (Aβ42), Auditory dysfunction, Auditory neuropathy, 5XFAD mouse model, Neural desynchronization, ABR

## Abstract

**Background:**

Emerging evidence suggests auditory dysfunction may serve as an early biomarker of Alzheimer’s disease (AD). This study investigates amyloid-beta 42 (Aβ42) accumulation in the cochlea and its relationship to auditory dysfunction in 5XFAD mice.

**Methods:**

Immunofluorescence imaging assessed Aβ42 deposition in cochlear structures (spiral ganglion neurons [SGNs], vasculature) at 8 weeks. Auditory brainstem responses (ABR) were analyzed using multimetric methods (Wave I amplitude, signal-to-noise ratio [SNR], phase-locking precision, cross-correlation) at 8 and 16 weeks.

**Results:**

Aβ42 deposition was detected in SGNs and vasculature by 8 weeks. 5XFAD mice exhibited reduced ABR Wave I amplitude (p < 0.01) and SNR versus wild-type, indicating impaired neural encoding. By 16 weeks, Wave I amplitude merged with cochlear microphonics, reflecting advanced neural deterioration. Synchrony analyses confirmed progressive auditory nerve desynchronization.

**Conclusion:**

Cochlear Aβ42 accumulation correlates with progressive auditory neuropathy in AD models, highlighting its biomarker potential. Multimetric ABR reveals neural synchrony deficits precede threshold shifts, emphasizing the need for advanced auditory assessments.

## Introduction

Alzheimer’s disease (AD) is a progressive neurodegenerative disorder characterized by cognitive decline and memory impairment(1, 2). While traditionally viewed as a primarily cognitive disorder, emerging evidence suggests that sensory impairments, particularly auditory dysfunction, may serve as early biomarkers of AD(3–5). Hearing loss is emerging as an early biomarker and modifiable risk factor for AD(6). Epidemiological studies have consistently linked hearing impairment to a higher risk of cognitive decline, suggesting shared mechanisms between auditory dysfunction and neurodegeneration(7). Central auditory deficits in AD have been relatively well-studied, including impairments in speech perception and temporal processing(8, 9). However, the role of peripheral auditory structures, particularly the cochlea, in AD-related hearing loss remains underexplored. This study investigates amyloid-beta 42 (Aβ42) accumulation in the cochlea and its relationship to progressive auditory dysfunction in the 5XFAD mouse model of AD. Given the proximity of the auditory system to neural circuits vulnerable to amyloid pathology, understanding the peripheral contributions to AD-related auditory dysfunction is critical for early detection and intervention.

Previous studies have primarily focused on central auditory deficits, linking AD to impairments in speech perception, temporal processing, and auditory brainstem function(10, 11). Reports suggest that AD patients exhibit prolonged auditory brainstem response (ABR) latencies and reduced Wave I amplitude, indicating disruptions in cochlear nerve transmission and auditory processing (12, 13). While some studies propose that these deficits are secondary to central dysfunction, others suggest a direct pathological involvement of peripheral auditory structures(14, 15). A growing body of evidence supports the hypothesis that Aβ42 accumulation in the cochlea may disrupt auditory nerve function, leading to progressive hearing impairment(16, 17). However, the extent and consequences of Aβ pathology in the cochlea remain poorly understood.

The complex interplay between central and peripheral auditory deficits in AD presents a significant challenge in understanding the progression of hearing loss in this disease. Central auditory dysfunction has been well-documented in AD patients and animal models(18, 19). However, emerging research suggests that ABR testing may serve as a non-invasive biomarker for AD. Studies have demonstrated correlations between ABR abnormalities, such as prolonged latencies, reduced amplitudes, and cognitive decline(20, 21). Furthermore, ABR changes have been observed in preclinical AD models prior to overt hearing loss or cognitive symptoms, indicating its potential utility in early detection(22, 23). These findings support the growing recognition of ABR as a tool for diagnosing and monitoring neurodegenerative changes associated with AD.

With peripheral auditory contributions to Alzheimer’s disease still not fully elucidated, we investigated whether Aβ42 accumulation in the cochlea correlates with progressive auditory dysfunction in the 5XFAD mouse model(24). This model, which rapidly develops amyloid pathology, provides a unique opportunity to study both early and late-stage auditory deficits(24–26). Using immunofluorescence imaging, we assessed Aβ42 deposition in cochlear structures, including SGNs, auditory nerve fibers, and cochlear vasculature at 8 weeks. Given that ABR Wave I originate from auditory nerve excitation, we hypothesized that Aβ42 accumulation in the cochlea contributes to progressive auditory nerve dysfunction, leading to neural desynchronization

To comprehensively characterize the impact of cochlear amyloid pathology on auditory processing, we employed a multimetric ABR approach, incorporating Wave I amplitude, signal-to-noise ratio (SNR), and cross-correlation analysis(27, 28). These measures allowed us to evaluate threshold shifts, neural synchrony, and conduction efficiency across early (8 weeks) and late (16 weeks) stages of pathology. By linking cochlear amyloid pathology with functional outcomes, our study aims to clarify the role of peripheral auditory involvement in AD progression and its potential as an early diagnostic biomarker.

Understanding these mechanisms could provide crucial insights into how auditory dysfunction contributes to AD progression. Furthermore, our findings support the use of ABR testing as a promising biomarker for detecting early neurodegenerative changes associated with AD. By bridging central and peripheral auditory research in AD models, this study offers a more comprehensive view of disease impact on the auditory system while highlighting new avenues for early diagnosis and intervention strategies.

## Results

### Progressive Auditory Dysfunction in 5XFAD Mice Revealed by ABR Deficits

ABR recordings were conducted at 8, 16, and 32 kHz across stimulus intensities ranging from 90 to 10 dB SPL. However, for detailed analysis, ABR responses at 90 dB SPL were selected, as suprathreshold recordings provide critical insights into auditory nerve function and neural synchrony beyond simple threshold shifts.

**Figure 1:**
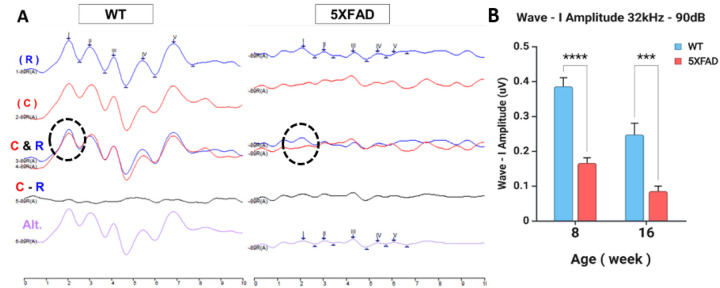
ABR Waveform Analysis and Wave I Amplitude Reduction in 5XFAD Mice at 32 kHz, 90 dB SPL. **(A)**Representative ABR waveforms recorded from a single mouse in each group (WT and 5XFAD) in response to rarefaction (R), condensation (C), and alternating polarity stimuli (Alt). The difference between condensation and rarefaction (C–R) illustrates group differences in neural synchrony and polarity sensitivity. Circles indicate Wave I peaks. **(B)** Mean Wave I amplitude values for WT and 5XFAD mice at 8 and 16 weeks. Data are presented as mean ± SEM. Statistical significance was determined using a two-way ANOVA followed by Bonferroni multiple comparisons test.

We analyzed ABR at 90 dB SPL, as it is sensitive to auditory dysfunction in AD models and humans, revealing reduced Wave I amplitude and prolonged latencies(20, 22). Suprathreshold ABR detects auditory deficits even when hearing sensitivity remains stable. Wave I amplitude at 8kHz and 16kHz did not show significant differences between 5XFAD and WT mice at 8 weeks, suggesting that high-frequency auditory deficits emerge earlier than mid- and low-frequency impairments in this model (supplementary Fig. 1). Based on these results, auditory dysfunction in 5XFAD mice was assessed by focusing on the basal region (32kHz) at 90 dB SPL. At 8 weeks, the morphological analysis of the ABR waves characteristics showed a clear difference WT and 5XFAD mice. WT mice exhibited robust Wave I amplitude, well-defined peaks, and high synchrony across rarefaction and condensation polarities (Fig. 1A).

Although rarefaction polarity typically elicits higher Wave I amplitudes, in the present study, condensation responses were unexpectedly higher in 5XFAD mice.

Condensation polarity (C) typically produces slightly delayed and reduced amplitudes. To isolate the cochlear microphonic (CM), we used C–R subtraction, which preserves hair cell responses while eliminating neural activity(29–31).

In WT mice, the C - R subtraction condition effectively eliminated cochlear microphonic (CM) components, confirming that the response was primarily neural in origin. In contrast, in 5XFAD mice, Wave I amplitude was significantly reduced, with blunted and desynchronized waveforms, indicating impaired auditory nerve transmission. The C - R subtraction failed to cancel cochlear microphonic contributions, suggesting an altered cochlear-neural relationship, possibly due to synaptic dysfunction or impaired phase-locking of the auditory nerve. Additionally, alternating polarity responses in 5XFAD mice appeared distorted, with increased waveform variability and noise contamination, reflecting reduced neural fidelity and potential hyperactivity within central auditory pathways (Fig. 1A).

A two-way ANOVA revealed a highly significant main effect of age (F_1, 18_ = 21.997, p = 0.00018), indicating that progression from 8 to 16 weeks had a robust impact on Wave I amplitude (Fig. 1B). There was also a significant main effect of strain (F_1, 18_ = 67.15, p < 0.0001), showing clear differences between WT and 5XFAD groups across ages. However, the interaction effect between age and strain was not significant (F_1, 18_ = 1.52, p = 0.234), suggesting that the age-related changes occurred in a similar direction across both genotypes. To explore specific group differences, a Bonferroni post hoc test was performed. Notably, at 8 weeks, 5XFAD mice showed a significantly altered response compared to WT (adjusted p < 0.0001). A similar pattern was observed at 16 weeks (adjusted p = 0.00075), reinforcing the genotype-driven divergence.

### Loss of Neural Synchrony in 5XFAD Mice Revealed by Cross-Correlation Deficits

**Figure 2:**
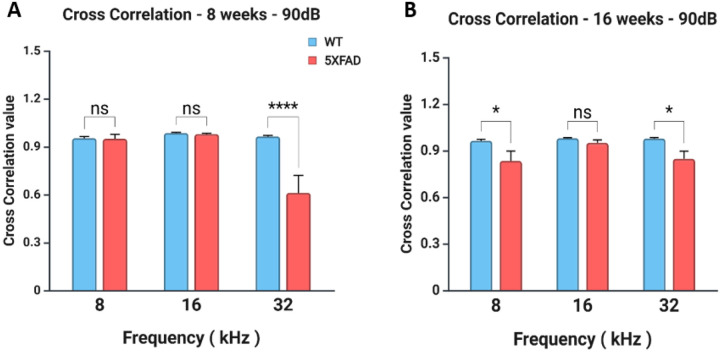
Cross-correlation values for WT (control) and 5XFAD mice at 8 weeks and 16 weeks under 90 dB stimulation. **(A)**Cross-correlation values at 8 weeks across 8, 16, and 32 kHz. WT mice showed consistently high cross-correlation values across frequencies, while 5XFAD mice exhibited a significant reduction, particularly at 32 kHz. Two-way ANOVA revealed a significant interaction effect (p < 0.0001) and a main effect of frequency (p < 0.0001). **(B)**Cross-correlation values at 16 weeks across the same frequencies. Although 5XFAD mice continued to show reduced cross-correlation values at 32 kHz, the interaction effect was not significant (p = 0.2551), and the main effect of frequency was also not significant (p = 0.1493), suggesting that the deficits observed at 8 weeks did not progress further by 16 weeks.

Cross-correlation analysis was performed across frequencies (8, 16, and 32 kHz) at 90 dB in WT and 5XFAD mice at 8 and 16 weeks to assess temporal coding fidelity in the auditory pathway, as deficits in synchrony may emerge in a frequency-dependent manner in neurodegenerative models (Fig. 2). At 8 weeks, two-way ANOVA revealed a significant main effect of frequency (F_2,51_ = 13.97, p < 0.0001) and a significant genotype × frequency interaction (F_2,51_ = 12.65, p < 0.0001) (Fig. 2A). Bonferroni post hoc comparisons indicated a significant reduction in cross-correlation in 5XFAD mice compared to WT at 32 kHz (adjusted p < 0.0001), while no differences were found at 8 or 16 kHz (adjusted p = 1.000 for both). At 16 weeks, two-way ANOVA showed a significant main effect of genotype (F_1,18_ = 12.22, p = 0.0026), with no significant main effect of frequency (F_2,18_ = 2.12, p = 0.1493) or interaction (F_2,18_ = 1.48, p = 0.2551). Post hoc analysis showed significantly lower cross-correlation values in 5XFAD mice at both 8 kHz (adjusted p = 0.0431) and 32 kHz (adjusted p = 0.0412), but not at 16 kHz (adjusted p = 1.000) (Fig. 2B). Together, these findings indicate a progressive and frequency-specific impairment in temporal coding in the 5XFAD model, with deficits emerging early at high frequencies and expanding to lower frequencies by 16 weeks.

### Progressive Auditory Dysfunction in 5XFAD Mice is Driven by Neural Desynchronization and Reduced Signal-to-Noise Ratio

Disruptions in neural synchrony suggest that 5XFAD mice exhibit greater temporal variability in auditory nerve responses, potentially contributing to degraded signal transmission. However, auditory dysfunction in AD is not solely due to desynchronization; signal reliability is equally important for effective neural encoding. To further assess the integrity of auditory processing, we analyzed residual noise (cochlear microphonic, CM) and signal-to-noise ratio (SNR), which measure how well the auditory system differentiates meaningful neural responses from background noise(10). Additionally, the relative contribution of CM vs. neural activity in 5XFAD mice was analyzed to determine whether increased residual noise could be masking neural responses, further exacerbating auditory deficits.

**Figure 3:**
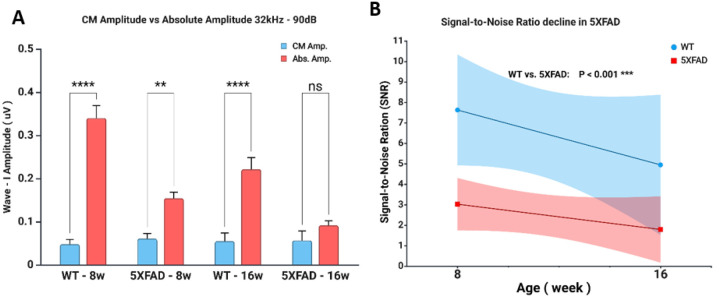
Severe Neural Desynchronization in 5XFAD Mice. **(A)** Cochlear microphonic (CM) amplitude (blue) and ABR Wave I absolute amplitude (red) recorded at 32 kHz, 90 dB SPL in WT and 5XFAD mice at 8 and 16 weeks. At 8 weeks, the difference between CM and absolute amplitude was significant, indicating that Wave I represent a distinguishable neural response. By 16 weeks, Wave I amplitude had further declined in 5XFAD mice and was nearly equivalent to CM amplitude, suggesting that the recorded signal is primarily residual cochlear microphonic activity embedded in background noise rather than a neural response. **(B)** SNR measured in WT and 5XFAD mice at 8 and 16 weeks. SNR remained significantly lower in 5XFAD mice at both ages, with a progressive decline over time. By 16 weeks, SNR in 5XFAD mice reached near baseline noise levels, indicating severe neural desynchronization and an inability to reliably detect Wave I as a distinct neural response.

To assess early alterations in peripheral auditory responses and signal fidelity in the 5XFAD model, we measured CM and calculated SNR at 32 kHz and 90 dB in WT and 5XFAD mice at 8 and 16 weeks (Fig. 3). Cochlear Microphonic was measured using alternating condensation and rarefaction polarities to cancel stimulus artifact and isolate receptor potential.

Two-way ANOVA on amplitude data revealed significant main effects of amplitude type (F_1,54_ = 106.85, p < 0.0001) and age (F_3,54_ = 14.35, p < 0.0001), as well as a significant amplitude × age interaction (F_3,54_ = 17.35, p < 0.0001). Bonferroni post hoc comparisons showed that absolute amplitudes were significantly greater than CM amplitudes at all time points and genotypes, with the largest differences observed in WT mice at 8 weeks (adjusted p < 0.0001) and progressively smaller differences in 5XFAD mice at 16 weeks (adjusted p = 0.0171) (Fig. 3A).

Linear regression analysis of SNR values revealed a significant difference in y-intercepts between WT and 5XFAD groups (F_1,23_ = 14.70, p = 0.0008), indicating that 5XFAD mice exhibited consistently lower SNR values across ages. No significant difference in slope was observed within either group (WT: F_1,11_ = 1.82, p = 0.2043; 5XFAD: F_1,11_ = 1.73, p = 0.2147), suggesting that the observed reduction in SNR reflects an early and sustained deficit in auditory signal encoding in 5XFAD mice (Fig. 3B). These results suggest that early-stage auditory dysfunction in 5XFAD mice is characterized by reduced Wave I amplitude, but by 16 weeks, the response becomes fully embedded in background noise, indicative of profound neural desynchronization.

### Polarity-Dependent ABR Distortions Reveal Neural Synchrony Deficits in 5XFAD Mice

The progressive auditory dysfunction observed in 5XFAD mice, characterized by reductions in ABR Wave I amplitude and neural synchrony, suggests significant impairments in auditory nerve transmission. However, these deficits alone do not fully capture the extent of auditory processing abnormalities, particularly in relation to phase-locked activity and polarity-dependent neural encoding. Since auditory neurons rely on precise timing mechanisms to encode temporal features of sound, disruptions in their ability to phase-lock to different stimulus polarities may further contribute to degraded auditory signal processing. To investigate this, we next analyzed polarity-dependent ABR responses in 5XFAD mice, examining whether alternating, condensation, and rarefaction stimulus conditions differentially impact auditory nerve function in this model.

**Figure 4:**
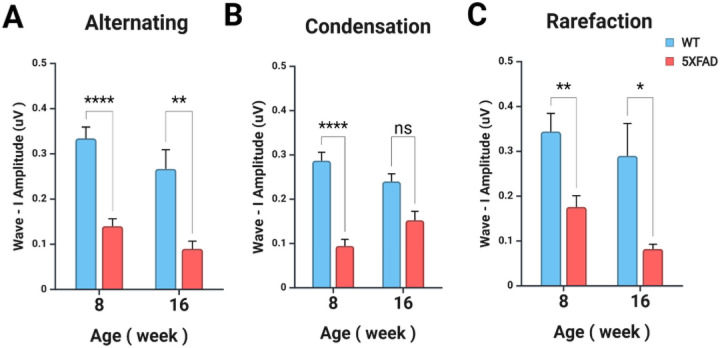
Wave I amplitudes measured under alternating, condensation, and rarefaction stimulus polarities in WT and 5XFAD mice at 8 and 16 weeks. **(A)** Wave I amplitude measured using alternating polarity stimuli at 32 kHz and 90 dB. **(B)**Wave I amplitude measured using condensation polarity. **(C)** Wave I amplitude measured using rarefaction polarity. In all stimulus conditions, 5XFAD mice exhibited lower amplitudes compared to WT, with further reductions observed at 16 weeks relative to 8 weeks. Data are represented as mean ± SEM. Statistical analysis performed using two-way ANOVA with Bonferroni post hoc test. *p <0.05, **p < 0.01, ****p < 0.0001.

Wave I amplitude (μV) was measured in response to a 32 kHz, 90 dB stimulus under three stimulus polarities: Alternating, Condensation, and Rarefaction (Fig. 4). Overall, 5XFAD mice exhibited reduced responses across all stimulus conditions, reflecting auditory dysfunction. For the Alternating condition, two-way ANOVA revealed no significant interaction between genotype and age (F_1,23_ = 0.093, p = 0.7637). The effect of age alone was not significant (F_1,23_ = 4.241, p = 0.05095). Amplitudes were greater in WT mice than in 5XFAD mice at both 8 and 16 weeks, with both groups showing a decline over time (Fig. 4A). In the Condensation condition, a significant interaction between genotype and age was observed (F_1,22_ = 5.607, p = 0.0270), indicating that the effect of age differed between genotypes. However, the main effect of age alone was not significant (F_1,22_ = 0.062, p = 0.8055). At both time points, amplitudes remained higher in WT mice, whereas 5XFAD mice exhibited a more pronounced age-related decline (Fig. 4B). For the Rarefaction condition, there was no significant interaction (F_1,23_ = 0.193, p = 0.6643) or main effect of age (F_1,23_ = 2.695, p = 0.1142). A similar pattern was observed, with WT mice consistently showing higher amplitudes than 5XFAD mice, and both groups demonstrating a decline at 16 weeks (Fig. 4C). Overall, these findings demonstrate that 5XFAD mice exhibit significantly reduced Wave I amplitudes compared to WT mice across all stimulus conditions, suggesting impaired auditory nerve function.

### Hippocampal and Cortical Amyloid Accumulation in 5XFAD Mice

To further investigate the relationship between amyloid deposition and potential functional deficits, we examined Aβ42 accumulation in key brain regions of 5XFAD mice, including the hippocampus, cortex, cerebellum, and thalamic sensory relay nuclei. Aβ42 immunohistochemistry (IHC) was employed to assess the extent and distribution of amyloid pathology, particularly in regions involved in auditory, motor, and cognitive processing. Given the reported involvement of vascular amyloid pathology, including cerebral amyloid angiopathy (CAA), in neurodegenerative changes affecting both central and peripheral systems, we also examined Aβ42 deposition along cerebrovascular structures. Prior studies indicate that Aβ42 accumulation in the auditory pathway correlates with ABR abnormalities, highlighting its potential role in auditory processing deficits in AD models (24, 28). This analysis aims to determine whether amyloid accumulation contributes to neural dysfunction and whether the presence of amyloid in sensory processing regions correlates with observed deficits in 5XFAD mice.

**Figure 5:**
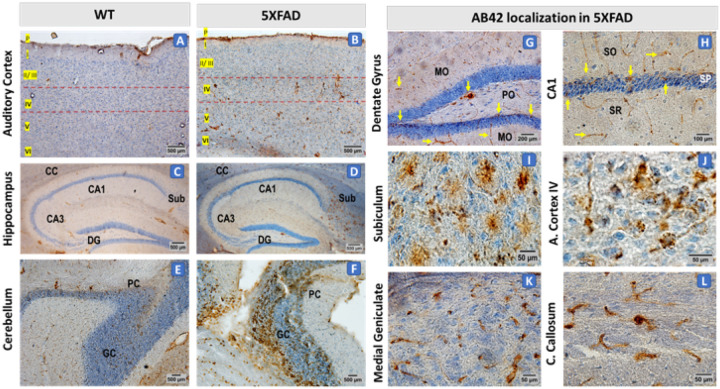
Aβ42 Immunohistochemistry Reveals Widespread Amyloid Deposition in the Brains of 16-Week-Old 5XFAD Mice. Representative Aβ42 immunohistochemical DAB staining in various brain regions of 16-week-old WT and 5XFAD mice. Sections were counterstained with hematoxylin. **(A-B)** In the auditory cortex, Aβ42 staining was minimal in WT controls (A), while 5XFAD mice (B) displayed prominent signal localized to layer IV, including vascular-associated deposition along the pia mater. **(C-D)** In the hippocampus, Aβ42 immunoreactivity was minimal in the WT controls (C), while Aβ42 localization was observed in the CA1, CA3, and dentate gyrus subfields, and the subiculum (D) in the 5XFAD mice. **(E-F)** Cerebellar sections from WT mice (E) showed faint or no signal, while 5XFAD mice (F) presented marked deposition in the molecular and Purkinje cell layers, extending into the granule cell layer. **(G-L)**High-magnification views highlight distinct cellular and extracellular Aβ42 distribution patterns in 5XFAD mice. (G) Dentate gyrus, dense, punctate extracellular Aβ42 deposits were observed within the molecular layer (MO) and polymorphic layer (PO), as indicated by arrows. **(H)** CA1 region, plaques are concentrated in stratum oriens (SO), stratum pyramidale (SP), and stratum radiatum (SR), as indicated by arrows. **(I)** High magnification in the subiculum, showed dense plaques with extracellular Aβ42 deposits. **(J)**The auditory cortex exhibited both cellular and extracellular Aβ42 staining. **(K)**The medial geniculate body showed scattered dense-core plaques. **(L)** The corpus callosum displayed Aβ42-positive staining associated with elongated, linear structures and smaller, rounded cells. Scale bars: 500 μm (A-F), 200 μm (G), 100 μm (H), 50 μm (I-L).

Immunohistochemical analysis revealed distinct Aβ42 staining patterns across multiple brain regions in 16-week-old 5XFAD mice compared to wild-type controls. In the hippocampus, moderate Aβ42 immunoreactivity was observed in the CA1, CA3, and dentate gyrus subfields, whereas the subiculum exhibited robust staining (Fig. 5A, B). In the auditory cortex, stronger Aβ42 immunoreactivity was localized to layer IV (Fig. 5C, D). Cerebellar sections displayed strong Aβ42 immunoreactivity in the molecular and Purkinje cell layers, with intense staining of Purkinje neurons and high Aβ42 accumulation in the granular layer (Fig. 5E, F). Higher magnification Aβ42 immunohistochemistry revealed distinct cellular and extracellular staining patterns. In the subiculum (Fig. 5G), dense, punctate extracellular Aβ42 deposits were observed. The cortex exhibited both cellular and extracellular Aβ42 staining, while the corpus callosum displayed Aβ42-positive staining associated with elongated, linear structures and smaller, rounded cells (Fig. 5J, I). Vascular-associated Aβ42 staining was evident along the pia mater of the cortex (Fig. 5H) and within blood vessel walls of the corpus callosum (Panel I). Cortical sections from WT mice showed no significant Aβ42 staining, whereas sections from 5XFAD mice showed dense amyloid accumulation along the pia mater and within deeper cortical layers, confirming a significant increase in Aβ42 burden in this region.

The presence of Aβ42-positive deposits near the cortical surface in 5XFAD mice suggests a potential interaction between amyloid plaques and cortical vasculature, reinforcing the hypothesis that amyloid accumulation disrupts neurovascular integrity in AD.

### Localization of Aβ42 in the cochlea

The cochlear vasculature shares properties with the cerebral microcirculation, making it a potential site for amyloid deposition. If Aβ42 infiltrates the cochlea via vascular transport or perilymphatic routes, it may contribute to cochlear dysfunction, mirroring vascular amyloid pathology in the brain. This mechanism is supported by studies demonstrating that cerebrovascular amyloid pathology disrupts the blood-brain barrier, leading to systemic Aβ42 accumulation in peripheral sensory structures. Examining Aβ42 accumulation in cochlear structures such as the stria vascularis and spiral ganglion will determine whether amyloid pathology extends to the peripheral auditory system and whether cochlear Aβ42 is linked to neurodegenerative processes observed in AD. This analysis could provide insights into AD’s systemic nature and potential early biomarkers.

To investigate Aβ42 localization in the cochlea, cryosectioned cochlear tissue from 5XFAD and WT mice at 8 weeks was stained with an anti-Aβ42 antibody and imaged using super-resolution Airyscan confocal microscopy.

**Figure 6:**
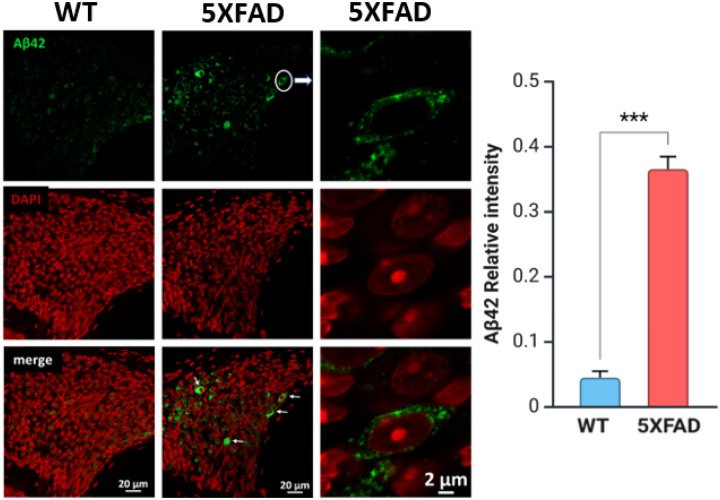
Aβ42 localization in the spiral ganglion neurons (SGNs) of 5XFAD and WT control mice at 8 weeks. Immunofluorescence images of cochlear cryosections stained for Aβ42 (green) and DAPI (red) reveal significant Aβ42 accumulation in SGNs of 5XFAD mice, while WT control mice exhibit minimal Aβ42 immunoreactivity. High-magnification images highlight intracellular Aβ42 deposits in SGNs of 5XFAD mice, with a punctate distribution in the perinuclear cytoplasmic region, White arrows indicate Aβ42-positive intracellular aggregates within SGNs. The quantification graph on the right shows significantly higher Aβ42-fluorescence intensity in SGNs of 5XFAD mice compared to controls (p < 0.001). Scale bars: 20 μm (low-magnification), 2 μm (high-magnification insets).

Immunofluorescence analysis revealed that 5XFAD mice exhibit significant intracellular Aβ42 accumulation in the spiral ganglion neurons (SGNs), whereas age-matched WT control mice show minimal Aβ42 immunoreactivity (Fig. 6). Higher magnification imaging confirmed a punctate distribution pattern of Aβ42 in the perinuclear cytoplasmic region of SGNs. DAPI staining showed well-defined nuclear morphology, with merged images displaying partial co-localization of Aβ42 with SGN nuclei. In contrast, WT control mice exhibited negligible Aβ42 staining, indicating a lack of amyloid pathology in SGNs. Quantification of relative fluorescence intensity demonstrated a significantly elevated Aβ42 burden in the 5XFAD group compared to controls Aβ42 localization. Given that phase-locked neural firing is essential for encoding temporal auditory cues, amyloid-associated axonal pathology could contribute to the observed neural desynchronization and reduced ABR synchrony in 5XFAD mice.

**Figure 7:**
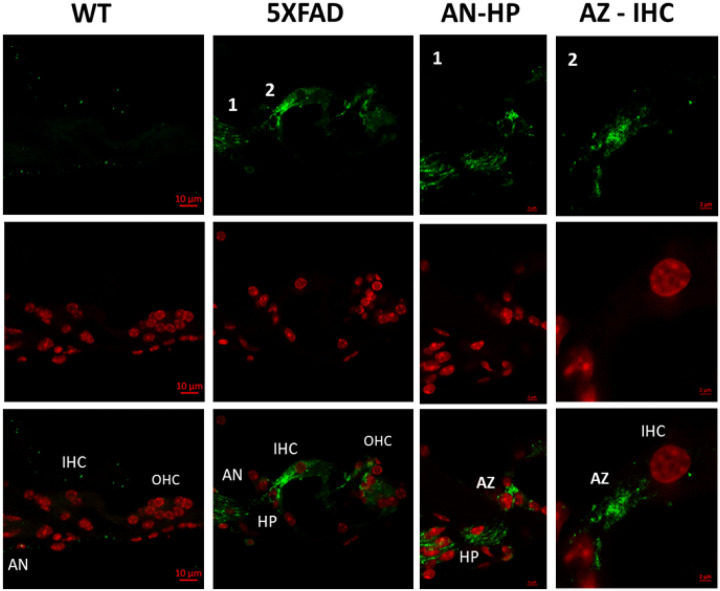
Aβ42 localization in the auditory nerve and organ of Corti of 5XFAD mice at 8 weeks. Confocal images show Aβ42 staining (green) and DAPI nuclear staining (red) in cochlear structures. In 5XFAD mice, Aβ42 accumulates in the inner hair cells (IHC) and outer hair cells (OHC), with distinct localization in the active zones (AZ) of ribbon synapses and the habenula perforata (HP), where auditory nerve (AN) fibers pass through the basilar membrane. High-magnification images confirm punctate Aβ42 deposits at the IHC-AZ interface, suggesting potential synaptic dysfunction. In contrast, WT control mice exhibit minimal Aβ42 immunoreactivity. Scale bars: 10 μm (low-magnification images), 1 μm (high-magnification insets)

Since SGNs showed Aβ42 accumulation, we next examined the organ of Corti to assess whether sensory and synaptic structures are also affected. Inner hair cells transmit auditory signals to SGNs via ribbon synapses, where disruptions can impair neural synchronization in the auditory nerve and Organ of Corti. Aβ42 localization in the active zones and habenula perforate could indicate early synaptic dysfunction, potentially contributing to auditory deficits in AD. Immunofluorescence analysis of the organ of Corti in 5XFAD mice at 8 weeks reveals significant Aβ42 accumulation in key auditory structures, while WT control mice exhibit minimal amyloid deposition (Fig. 7).

Aβ42 immunoreactivity is observed in the region of the inner hair cells, outer hair cells, and the auditory nerve, suggesting potential amyloid involvement in cochlear synaptic and neural processing. High-magnification images confirm Aβ42 deposition in the active zone of the inner hair cell ribbon synapse, an essential site for neurotransmitter release to auditory nerve fibers.

The presence of Aβ42 within the active zones of ribbon synapses suggests potential disruption of synaptic vesicle cycling, a critical process for sustained neurotransmitter release at the IHC-SGN interface. This aligns with evidence that amyloid pathology impairs vesicle docking and synaptic integrity in other central nervous system regions(32). Such deficits may contribute to polarity-dependent ABR amplitude reductions observed in 5XFAD mice, as impaired synaptic transmission could selectively alter rarefaction and condensation responses The immunolabeling of Aβ42 in the habenula perforata region, where auditory nerve fibers pass through the basilar membrane, suggests possible amyloid-related axonal pathology that may contribute to neural desynchronization and impaired auditory transmission.

#### Aβ42 accumulates in the strial microvasculature

In 5XFAD mice, Aβ42 deposits are localized primarily within the blood vessels (BV) of the stria vascularis, with high-magnification images confirming punctate Aβ42 accumulation in endothelial cells (EC). Marginal cells (MC), intermediate cells (IC), and basal cells (BC) show no apparent Aβ42 accumulation, suggesting a selective vulnerability of the vascular compartment to amyloid deposition. The distribution pattern indicates that Aβ42 is predominantly associated with the endothelial lining of the microvasculature, suggesting potential blood-labyrinth barrier disruption or vascular amyloid pathology similar to cerebral amyloid angiopathy. In contrast, WT mice exhibit no detectable Aβ42 accumulation in the stria vascularis, indicating an absence of amyloid pathology under non-transgenic conditions. These findings suggest early vascular involvement in amyloid pathology within the cochlea, which may contribute to microvascular dysfunction and impaired cochlear homeostasis in 5XFAD mice.

## Discussion

We provide direct evidence of Aβ42 accumulation in the 5XFAD mouse cochlea at 8 weeks, supporting its role in early auditory dysfunction in AD. Immunofluorescence revealed intracellular Aβ42 in spiral ganglion neurons (SGNs), the auditory nerve, and cochlear vasculature. Its presence in SGNs and ribbon synapses suggests synaptic impairment, likely contributing to ABR Wave I reductions. Furthermore, the presence of Aβ42 in cochlear blood vessels raises the possibility of a vascular contribution to peripheral auditory dysfunction, similar to amyloid-related microvascular pathology in the brain. These findings align with recent studies indicating that amyloid-related disturbances in the cochlear perilymph may disrupt inner ear homeostasis and contribute to auditory deficits in Alzheimer’s disease models(33). The detection of Aβ and tau proteins in human perilymph further strengthens the hypothesis that amyloid pathology extends beyond the central nervous system and into the peripheral auditory structures(17).

The presence of Aβ42 in the cochlea has also been demonstrated by Omata et al.(16), who used a different approach by establishing transgenic mouse lines that specifically express Aβ42 or its derivative in cochlear hair cells, rather than using a brain-targeted AD model. Their findings suggest that direct Aβ42 expression in hair cells can independently lead to early-onset auditory dysfunction, particularly in high-frequency hearing loss. In contrast, our study utilized a conventional AD transgenic model (5XFAD) with systemic amyloid pathology, demonstrating that Aβ42 deposition in the cochlea occurs even in the absence of cochlea-specific genetic modifications. This distinction is important because it suggests that Aβ42 accumulation in the cochlea is not an isolated phenomenon but part of the broader neurodegenerative process in Alzheimer’s disease models.

Although this study confirms Aβ42 accumulation in the cochlea at 8 weeks, immunohistochemical analysis was not extended to 16 weeks, as the primary objective was to characterize functional auditory deficits over time rather than track Aβ42 deposition at multiple time points. Prior work has demonstrated that cerebrospinal fluid (CSF) dynamics play a crucial role in amyloid clearance from the cochlea, and impaired glymphatic function has been implicated in the accumulation of Aβ in neurodegenerative conditions(34). Given that ABR Wave I amplitude and signal-to-noise ratio (SNR) decline by 16 weeks despite stable cochlear microphonic (CM) responses, the data suggest that neural desynchronization—rather than increasing cochlear amyloid burden—is the predominant driver of late-stage auditory impairment. Additionally, studies have shown that Aβ clearance from the cochlea may occur through CSF pathways, further emphasizing the importance of cerebrovascular and central mechanisms in late-stage auditory dysfunction(35). Given the role of the glymphatic system in Aβ clearance, its dysfunction may contribute to progressive neural desynchronization, a hallmark of auditory neuropathy in AD models.

While Aβ42 accumulation in the cochlea provides evidence of peripheral amyloid pathology, its functional consequences on auditory processing remain critical to understanding how Alzheimer’s disease affects hearing. Given that Aβ42 was detected in spiral ganglion neurons and ribbon synapses, its presence may contribute to early synaptic dysfunction, potentially impairing auditory nerve conduction. To assess the impact of these pathological changes, ABR recordings were analyzed using a multimetric approach, incorporating waveform morphology, Wave I amplitude, phase-locking analysis, and SNR to comprehensively evaluate auditory neural function and characterize the extent of auditory processing deficits in 5XFAD mice.

The significant reduction in ABR Wave I amplitude in 5XFAD mice suggests early auditory nerve dysfunction, likely due to synaptic deficits at the inner hair cell-auditory nerve junction. Since Wave I reflects cochlear nerve excitation, its decline aligns with synaptopathy models of AD, where amyloid accumulation disrupts synaptic integrity. (36–38). These findings are consistent with previous studies showing cochlear nerve dysfunction as an early marker of AD-related auditory decline(39). The continued reduction at 16 weeks indicates progressive auditory neuropathy, suggesting that synaptic degradation worsens over time, leading to reduced neural recruitment. Given the parallels between these findings in 5XFAD mice and human AD patients, ABR measures may have potential as a non-invasive biomarker for early disease detection (40). Identifying ABR abnormalities before significant cognitive symptoms arise could aid in earlier diagnosis and intervention, especially in populations at high risk for AD.

Further supporting the hypothesis of progressive auditory nerve dysfunction is the persistence of cochlear microphonic (CM) activity despite the loss of Wave I amplitude. CM originates primarily from the outer hair cells and remains intact in auditory neuropathy spectrum disorders, even when neural synchrony is lost(41). At 8 weeks, Wave I amplitude in 5XFAD mice remained distinct from CM amplitude, indicating that neural responses were still present. However, by 16 weeks, Wave I amplitude was no longer distinguishable from CM, suggesting that the recorded signal primarily reflects residual cochlear microphonic activity rather than a true neural response(42). This finding is consistent with reports that CM can persist in cases of severe auditory nerve impairment, often leading to misinterpretation in standard audiological assessments(30). The persistence of CM despite neural degeneration reinforces the idea that amyloid pathology targets auditory nerve synchrony rather than sensory receptor function.

While neural synchrony was compromised, SNR analysis further quantified the reliability of auditory responses over time. SNR values in 5XFAD mice were significantly lower than in WT at 8 weeks, but the difference became more pronounced at 16 weeks, when SNR declined to baseline noise levels. Low SNR is a known indicator of neural desynchronization and impaired auditory processing, commonly observed in neurodegenerative conditions affecting auditory nerve transmission(43). Given that cochlear microphonics remained stable while SNR declined, this suggests that auditory nerve conduction is severely compromised, leading to a near-complete loss of phase-locked neural activity. Similar patterns of prolonged CM duration and reduced ABR amplitudes have been observed in human Alzheimer’s disease patients, reinforcing the hypothesis that early auditory processing deficits in AD result from progressive neural desynchronization rather than peripheral sensory dysfunction (44).

The progressive loss of auditory nerve synchrony in 5XFAD mice is further supported by deficits in cross-correlation and phase-locking, both of which indicate disrupted neural coordination across the auditory pathway. Cross-correlation analysis revealed a significant reduction in neural synchrony, particularly at 16 weeks, suggesting that the ability of auditory neurons to fire in a coordinated manner deteriorates over time. This decline in cross-correlation values mirrors the patterns observed in human AD patients, where phase-locked neural activity is impaired, leading to difficulties in speech perception and sound localization (45–47). The observed reduction in phase-locking, particularly in later ABR waves (Waves III and V), suggests that amyloid pathology disrupts temporal processing at multiple levels of the auditory system. Since Wave V originates in the inferior colliculus, this finding points to deficits in midbrain auditory circuits, which are crucial for integrating auditory information and relaying it to cortical regions. The decline in phase-locking precision over time suggests that amyloid-driven neurodegeneration affects not only the auditory nerve but also downstream auditory processing centers including inferior colliculus, medial geniculate body and auditory cortex, leading to widespread neural desynchronization.

These physiological abnormalities are further compounded by polarity-dependent ABR distortions, which provide additional evidence of synaptic impairment. The disproportionately greater reduction in rarefaction responses compared to condensation responses suggests impaired synaptic transmission at the inner hair cell-auditory nerve junction. Rarefaction stimuli elicit stronger basilar membrane displacement, which typically leads to greater neural activation. The selective vulnerability of rarefaction responses in 5XFAD mice is consistent with findings in auditory neuropathy spectrum disorders, where ribbon synapse loss leads to deficits in phase-locked neural activity(45, 46, 48–50). This pattern of impairment suggests that amyloid-driven synaptopathy disrupts the early stages of auditory signal encoding, potentially interfering with the fidelity of neural responses to complex sounds. By 16 weeks, rarefaction and alternating polarity responses exhibited the greatest reductions, further supporting the idea that amyloid pathology contributes to progressive neural desynchronization in Alzheimer’s disease.

Compared to WT controls, 5XFAD mice exhibited significant amyloid deposition in key brain regions, including the subiculum, cortical layers (particularly layer IV of the auditory cortex), and sensory processing regions such as the medial geniculate body of the thalamus. This widespread amyloid pathology reinforces the potential role of Aβ42 accumulation in both cognitive and auditory deficits in this model. The prominence of vascular amyloid pathology, particularly along the pia mater of the cortex, further supports the hypothesis that cerebrovascular dysfunction contributes to neurodegeneration in both cognitive and sensory systems. Given that vascular amyloid accumulation has been linked to disruptions in the blood-brain barrier and impaired metabolic support for neurons, it is plausible that similar mechanisms affect auditory pathways(51, 52). This could disrupt neural transmission within auditory circuits and increase their vulnerability to degeneration, potentially leading to the auditory processing deficits observed in 5XFAD mice. These findings raise the possibility that vascular amyloid accumulation may contribute to auditory dysfunction in Alzheimer’s disease, acting through mechanisms within the brain, by disrupting neural transmission and increasing the vulnerability of auditory circuits to degeneration.

ABR deficits in 5XFAD mice parallel those in human AD patients, suggesting its potential as a non-invasive biomarker. The persistence of cochlear microphonics despite neural decline underscores the need to distinguish sensory vs. neural contributions in early AD detection. Future research should investigate cochlear amyloid clearance, ABR-based SNR analysis, and therapeutic strategies for early auditory dysfunction.(53–55).

By 16 weeks, 5XFAD mice exhibit complete neural desynchronization, emphasizing the potential of ABR and SNR analysis for detecting early neurodegenerative changes. Future research should investigate whether early ABR deficits in 5XFAD mice predict later cognitive impairment, further strengthening the clinical relevance of these findings. Additionally, pharmacological interventions targeting synaptic preservation or amyloid clearance should be explored as potential therapeutic strategies to mitigate auditory and cognitive decline.

### Model Considerations and Translational Relevance

The 5XFAD mouse is a well-characterized and aggressive model of early-onset Alzheimer’s disease, engineered to overexpress human APP and PSEN1 mutations associated with familial AD. It exhibits early and robust Aβ42 accumulation, enabling investigation of amyloid-driven neurodegenerative processes, including auditory system vulnerability. This makes it a valuable tool for examining sensory circuit disruptions that precede overt cognitive decline. Notably, recent clinical evidence has demonstrated the presence of Aβ and tau in perilymph of aged cochlear implanting patients, with levels correlating with cognitive decline and age, supporting the relevance of using this model to explore auditory biomarkers of AD(17).

However, certain aspects of the 5XFAD model represent experimental artifacts rather than physiological disease progression. The use of the neuron-specific Thy1 promoter results in supraphysiological expression of mutant APP, which may not reflect endogenous regulation in human AD. Moreover, while amyloid plaques are a hallmark feature, the model lacks tauopathy and neurofibrillary tangles typical of late-stage human Alzheimer’s pathology. Additionally, the absence of comorbid factors such as vascular dysfunction and aging limits the generalizability of certain findings to sporadic AD. Thus, while 5XFAD provides valuable insight into early Aβ-related mechanisms, results should be interpreted with awareness of its engineered limitations.

## Conclusion

This study provides direct evidence of Aβ42 accumulation in the cochlea of 5XFAD mice, demonstrating that peripheral amyloid pathology may contribute to early auditory dysfunction in AD. Immunofluorescence analysis confirmed Aβ42 deposition in spiral ganglion neurons and cochlear vasculature, suggesting a potential role in disrupting synaptic transmission and neural synchrony. Using a multimetric ABR approach, we identified early reductions in Wave I amplitude and SNR at 8 weeks, followed by a profound neural desynchronization by 16 weeks, where Wave I responses were indistinguishable from background noise. These findings suggest a progressive auditory neuropathy-like dysfunction, reinforcing the hypothesis that neural synchrony deficits precede severe hearing impairment in AD models.

Our results emphasize the need to examine peripheral auditory pathology in AD, an area often overshadowed by research on central auditory deficits. By demonstrating early cochlear Aβ42 accumulation and its impact on auditory function, this study highlights a potential biomarker for early AD detection, and ABR Wave I amplitude and SNR, identified in this study, underscore the clinical potential of these non-invasive auditory metrics as early biomarkers for AD, with promising applications such as early detection of at-risk individuals and monitoring of treatment efficacy. Future research should determine whether cochlear amyloid pathology can serve as an early diagnostic marker and explore its potential for therapeutic targeting in AD-related auditory impairment. Furthermore, our continuing work aims to elucidate the impact of Aβ42 in the cochlea at the cellular level, specifically investigating its effects on synapse integrity, inflammation, and apoptosis, to provide a more comprehensive understanding of the mechanisms driving auditory dysfunction in AD.

## Material and methods

### Animals:

This study employed two mouse strains: 5XFAD transgenic mice and C57BL/6J (WT) controls (Jackson Laboratories, Bar Harbor, ME, USA). The 5XFAD model was selected for its accelerated Alzheimer’s disease (AD) pathology, enabling early investigation of Aβ42 accumulation and related neurophysiological deficits, including auditory dysfunction. The 5XFAD line carries five familial AD (FAD) mutations—three in the amyloid precursor protein (APP: K670N/M671L [Swedish], I716V [Florida], and V717I [London]) and two in presenilin-1 (PSEN1: M146L and L286V)—under the control of the neuron-specific Thy1 promoter. These mutations result in rapid Aβ42 overproduction, with detectable soluble Aβ42 in the brain as early as 4 weeks. By 6 weeks, intraneuronal accumulation becomes pronounced, with amyloid plaque formation in the cortex and subiculum by approximately 2 months. Beyond cognitive impairment, this model also exhibits early auditory deficits, including elevated ABR thresholds, reduced Wave I amplitude, and impaired temporal processing, such as gap detection. A total of 24 male mice (12 WT and 12 5XFAD) were used in this study. This sample size provides sufficient statistical power to detect genotype-dependent differences while adhering to ethical guidelines for animal research. Animals were housed in polycarbonate cages (3–4 mice per cage) under standard laboratory conditions: temperature of 22–24°C, 50–60% humidity, and a 12-hour light/dark cycle. Food and water were provided ad libitum. Routine health monitoring included daily welfare assessments and weekly weight tracking. Animals exhibiting > 10% weight loss within a week or signs of distress were excluded from further testing. All experimental procedures were approved by the Institutional Animal Care and Use Committee (IACUC) of Southern Illinois University School of Medicine and complied with the NIH Guide for the Care and Use of Laboratory Animals and ARRIVE (Animal Research: Reporting of In Vivo Experiments) guidelines. Humane endpoints were established, and every effort was made to minimize animal distress and ensure reproducibility. Mice were randomly assigned to experimental groups (WT or 5XFAD) upon arrival. All ABR recordings and histological analyses were conducted blind to genotype, with coded identifiers used during data acquisition and throughout the analysis process. Blinding was maintained until all data were finalized to ensure unbiased interpretation.

### Auditory Brainstem Response (ABR)

ABR recordings were performed to evaluate auditory nerve function and brainstem transmission in WT and 5XFAD mice. ABR waveforms reflect sequential neural activation along the auditory pathway, with Wave I representing the compound action potential (CAP) of the auditory nerve and spiral ganglion neurons, serving as a key indicator of peripheral auditory integrity. Animals were anesthetized with an intraperitoneal injection of ketamine (20 mg/ml) and xylazine (2 mg/ml) in 0.9% saline at a dose of 0.1 mL per 20 g body weight. Body temperature was maintained using a heating pad, and depth of anesthesia was monitored via the pedal reflex. Subdermal needle electrodes were positioned with the active lead at the vertex, reference behind the ipsilateral pinna, and ground at the contralateral mastoid or back. ABR testing was conducted in a sound-attenuated chamber using a closed-field speaker system inserted into the external auditory canal. Stimuli were generated via Intelligent Hearing Systems software and consisted of 5-ms tone bursts delivered at 50 stimuli per second with 512-sweep averaging. Stimuli were presented in 10 dB decrements from 90 dB SPL to 10 dB SPL across three frequencies—8, 16, and 32 kHz—corresponding to apical, middle, and basal cochlear regions, respectively. ABR signals were amplified 100,000×, filtered (0.1–3 kHz), and digitally sampled for analysis.

For waveform analysis, responses to rarefaction and condensation polarities were collected separately to isolate neural activity from cochlear microphonics (CM). Alternating polarity responses were also recorded to minimize stimulus artifacts. To enhance signal specificity, C–R subtraction was employed to isolate CM components. A minimum threshold of 0.1 μV Wave I amplitude was required to validate response detection. While thresholds were measured at all intensities, suprathreshold responses at 90 dB SPL were selected for detailed analysis, as they reveal early auditory nerve dysfunction prior to threshold elevation. This approach allows detection of neural desynchronization, altered latency, and reduced amplitude, features that are especially relevant to Alzheimer’s disease models. Cross-correlation analysis was conducted across trials to assess temporal fidelity and neural synchrony, with emphasis on frequency-specific deficits. Additionally, signal-to-noise ratio (SNR) and CM amplitude were quantified to evaluate auditory encoding reliability and differentiate neural signal degradation from elevated background activity.

### Tissue Fixation and Cryosectioning

Histological analysis was performed on brain hemispheres from 5xFAD and WT mice aged 16 weeks (n = 6). Following euthanasia with sevoflurane anesthesia, brains were rapidly extracted and bisected along the midline. Hemispheres were immediately transferred into ice-cold 4% paraformaldehyde (PFA) and fixed overnight at 4°C. The following day, tissues were cryoprotected by sequential immersion in 10% sucrose for 2 hours and 30% sucrose overnight at 4°C. Samples were then washed thoroughly with PBS and embedded in optimal cutting temperature (OCT) compound, frozen, and sectioned sagittally at 20 μm thickness using a cryostat. Sections were collected onto Superfrost Plus microscope slides and stored at − 20°C until further analysis.

Cochleae were extracted from temporal bones immediately after brain removal and post-fixed in 4% PFA for 2 hours at 4°C. Decalcification was performed by immersing cochleae in 0.12 M EDTA (pH 7.4) at 4°C for 72–96 hours, with gentle agitation and daily solution changes. Following decalcification, samples were cryoprotected in 10% sucrose for 2 hours and 30% sucrose overnight at 4°C. Cochleae were then embedded in OCT compound and stored at − 80°C until sectioning. Mid-modiolar sections (20 μm) were obtained using a cryostat and mounted on Superfrost Plus slides for subsequent immunostaining.

### Aβ42 validation and selectivity

To ensure specificity and validate amyloid-beta 42 (Aβ42) detection, two monoclonal antibodies targeting distinct epitopes were used in parallel. The first, β-Amyloid (1–42) (E6D5M) Rabbit mAb (#24090, Cell Signaling Technology), recognizes a unique C-terminal epitope specific to Aβ42. Its binding is highly selective for the free carboxyl group at alanine-42, distinguishing Aβ42 from shorter isoforms such as Aβ40. This antibody preferentially detects Aβ42 in its native or aggregated conformation, making it suitable for distinguishing full-length peptide accumulation in tissue. The second antibody, MO22142 (Neuromics), is a mouse IgG2a mAb that targets the N-terminal region (residues 1–16) common to multiple Aβ isoforms, including Aβ42, Aβ40, and Aβ38, but only after β-secretase cleavage. It does not bind unprocessed APP, as the required N-terminal epitope is inaccessible within the precursor. By combining these two antibodies—one specific to the C-terminus of Aβ42 and the other recognizing cleaved Aβ peptides—plaque identity and localization were independently confirmed. Parallel staining ensured signal validation and reduced the likelihood of false-positive detection from isoform overlap or precursor fragments.

### DAB-Based Immunohistochemistry

For chromogenic detection of Aβ42, frozen brain and cochlear sections were processed using the Millipore IHC Select^®^ Immunoperoxidase Secondary Detection System. After PBS washes, sections were permeabilized in methanol at − 20°C for 20 minutes. Endogenous peroxidase activity was quenched using 0.3% hydrogen peroxide in PBS for 10 minutes at room temperature. Sections were then blocked in PBS containing 5% normal donkey serum and 0.3% Triton X-100 for 1 hour. Primary staining was performed overnight at 4°C using rabbit anti-Aβ42 monoclonal antibody (clone E6D5M; Cell Signaling Technology, 1:400). The following day, sections were incubated sequentially with biotinylated secondary antibody, HRP-conjugated streptavidin, and developed using 3,3′-diaminobenzidine (DAB) substrate according to the manufacturer’s protocol. Slides were counterstained with hematoxylin, rinsed with deionized water, and coverslipped using ProLong^®^ Diamond Antifade Mountant (Invitrogen) before imaging under a light microscope.

### Immunofluorescence Staining

Cochlear sections were washed in PBS and permeabilized with ice-cold absolute ethanol for 10 minutes, then blocked for 1 hour in PBS containing 5% donkey serum and 0.3% Triton X-100. Primary antibodies against Aβ42—Rabbit IgG (clone E6D5M; Cell Signaling Technology, 1:400) and Mouse IgG2a (Neuromics MO22142, 1:1000)—were applied overnight at 4°C. Following PBS washes, sections were incubated with Alexa Fluor 568-conjugated goat anti-rabbit IgG (1:1000, Invitrogen) and Alexa Fluor 488-conjugated goat anti-mouse IgG (1:1000, Invitrogen, Cat# 21131) for 1 hour at room temperature. Nuclei were counterstained with DAPI. Slides were mounted with ProLong^®^ Diamond Antifade Mountant (Invitrogen) and imaged using confocal microscopy.

### Amylo – Glo / Plaques staining

To fluorescently label amyloid plaques, sagittal brain sections (20 μm) were stained using Amylo-Glo RTD^™^ Amyloid Plaque Stain Reagent (Biosensis, Cat# TR-400-AG), following the manufacturer’s protocol. Slides were first rinsed in PBS and incubated for 10 minutes in the Amylo-Glo working solution prepared in 0.9% saline. Sections were then washed again in PBS and counterstained with Ethidium Bromide, as recommended in the Amylo-Glo RTD^™^ kit, to enable nuclear visualization. Slides were mounted with ProLong^®^ Diamond Antifade Mountant (Invitrogen) and stored at 4°C in the dark until imaging. Amylo-Glo was selected for its high sensitivity, minimal background fluorescence, and compatibility with multi-channel confocal imaging, as previously validated in amyloid-rich Alzheimer’s disease models(56).

### Confocal images with Airyscan

Fluorescently labeled brain and cochlear sections were imaged using a Zeiss LSM 800 confocal microscope equipped with an Axio Observer.Z1/7 inverted stand and GaAsP-PMT detectors. High-resolution imaging was performed using EC Plan-Neofluar 40×/1.3 Oil and 63× oil immersion objectives, with frame scan mode and bidirectional scanning enabled. Detector gain was set to 850 V, with a digital gain of 1.0. Image acquisition was conducted under Airyscan SR (Super-Resolution) mode (2D auto) with an Airyscan strength setting of 10.0, and averaging was applied depending on signal intensity and background levels. The scan zoom was set to 0.8× for most overview images, while zoomed acquisitions were performed to visualize intracellular amyloid structures and subcellular localization. Imaging parameters, including laser power and detector settings, were held constant within experiments for comparative analysis. All image processing, intensity quantification, and profile analyses were performed using ZEN 3.0 software (Zeiss) with standardized LUTs and acquisition metadata.

### Statistical analysis

All statistical analyses were performed using GraphPad Prism 9 (GraphPad Software, San Diego, CA). Data are presented as mean ± SEM, and statistical significance was determined using two-way ANOVA followed by Bonferroni post hoc tests for multiple comparisons unless otherwise indicated. Specifically, when comparing Aβ42 DAB staining intensity in the brain between WT and 5XFAD mice, an unpaired t-test with Welch’s correction was used to account for potential unequal variances between groups. For waveform and polarity condition comparisons, interaction effects and main effects (genotype, age, frequency) were reported along with corresponding F values and p-values. Linear regression analysis was used to evaluate relationships between auditory measures such as SNR and age, with slopes and intercepts compared across genotypes. Significance was defined at p < 0.05, with adjusted p-values reported when applicable. Figures were organized and finalized using BioRender for visual clarity and stylistic consistency.

## Figures and Tables

**Figure 8:**
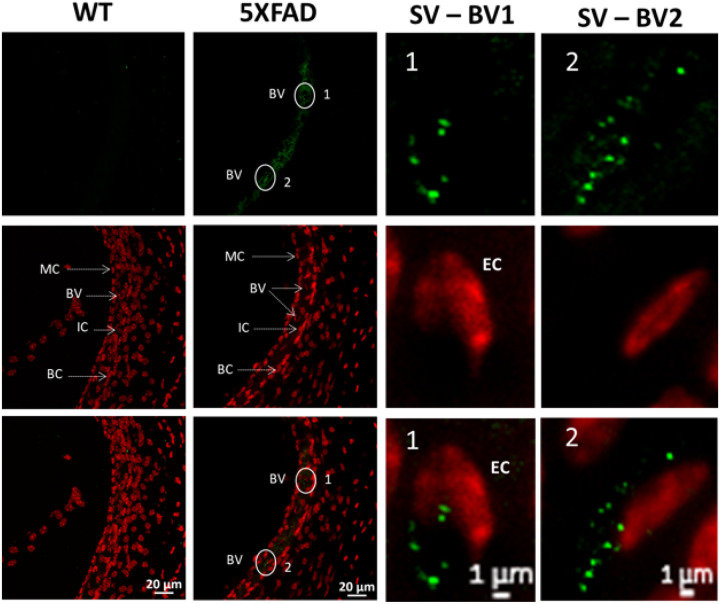
Aβ42 accumulation in the stria vascularis of 5XFAD and WT mice at 8 weeks. Representative immunofluorescence images of cochlear cryosections stained for Aβ42 (green) and DAPI (red) show significant Aβ42 deposition in the stria vascularis of 5XFAD mice, whereas WT controls exhibit minimal amyloid immunoreactivity. In 5XFAD mice, Aβ42 is primarily localized within blood vessels (BV), with high-magnification images confirming punctate Aβ42 accumulation in endothelial cells (EC). Marginal cells (MC), intermediate cells (IC), and basal cells (BC) do not exhibit apparent Aβ42 accumulation. The presence of Aβ42 within the vascular compartment suggests early vascular amyloid pathology, potentially contributing to blood-labyrinth barrier dysfunction. Scale bars: 20 μm (low-magnification images), 1 μm (high-magnification insets).

## Data Availability

Data is provided within the manuscript or supplementary information files
